# Effects of Intranasal Oxytocin on Pup Deprivation-Evoked Aberrant Maternal Behavior and Hypogalactia in Rat Dams and the Underlying Mechanisms

**DOI:** 10.3389/fnins.2019.00122

**Published:** 2019-02-26

**Authors:** Xiao Yu Liu, Dongyang Li, Tong Li, Haitao Liu, Dan Cui, Yang Liu, Shuwei Jia, Xiaoran Wang, Runsheng Jiao, Hui Zhu, Fengmin Zhang, Danian Qin, Yu-Feng Wang

**Affiliations:** ^1^Department of Physiology, School of Basic Medical Sciences, Harbin Medical University, Harbin, China; ^2^Department of Pathogen, School of Basic Medical Sciences, Harbin Medical University, Harbin, China; ^3^Department of Physiology, Shantou University of Medical College, Shantou, China

**Keywords:** pup deprivation, intranasal drug application, lactation, maternal health, oxytocin

## Abstract

Oxytocin (OT), a hypothalamic neuropeptide, applied through nasal approach (IAO), could improve maternal health during lactation that is disrupted by mother–baby separation; however, the regulation of IAO effects on maternal behaviors and lactation as well as the underlying mechanisms remain unclear. Using lactating rats, we observed effects of intermittent pup deprivation (PD) with and without IAO on maternal behaviors and lactation as well as the activity of OT neurons in the supraoptic nucleus (SON) and the activity of hypothalamic pituitary-adrenal axis, key factors determining the milk-letdown reflex during lactation and maternal behaviors. The results showed that PD reduced maternal behaviors and lactation efficiency of rat dams as indicated by significantly longer latency to retrieve their pups and low litter’s body weight gains during the observation, respectively. In addition, PD caused early involution of the mammary glands. IAO partially improved these changes in rat dams, which was not as significant as IAO effects on control dams. In the SON, PD decreased c-Fos and increased glial fibrillary acidic protein (GFAP) filaments significantly; IAO made PD-evoked c-Fos reduction insignificant while reduced GFAP filament significantly in PD dams. IAO tended to increase the levels of phosphorylated extracellular signal-regulated kinases (pERK) 1/2 in PD dams. Moreover, PD+IAO significantly increased plasma levels of dam adrenocorticotropic hormone and corticosterone but not OT levels. Lastly, PD+IAO tended to increase the level of corticotropin-releasing hormone in the SON. These results indicate that PD disrupts maternal behaviors and lactation by suppressing the activity of hypothalamic OT-secreting system through expansion of astrocytic processes, which are partially reversed by IAO through removing astrocytic inhibition of OT neuronal activity. However, the improving effect of IAO on the maternal health could be compromised by simultaneous activation of hypothalamic pituitary-adrenocortical axis.

## Introduction

Lactation is essential for maintaining the species of mammals and an irreplaceable factor for mental and physical health of mothers and the babies. However, lactation is vulnerable to many adverse factors, such as mother–baby separation (Wang and Hatton, 2009b), lacking social supports (Trickey and Newburn, 2014), obesity (Stuebe et al., 2014), babies’ sickness (Lawrence, 2013), poor breast conditions (Tang et al., 2013), cesarean section (Orun et al., 2010), mothers’ using drugs that are toxic to the babies (Varalda et al., 2012), early usages of bottle feeding and milk substitutes (Jiang et al., 2012), working requirements (Oslislo and Kaminski, 2000), and others (Seema et al., 1997; Berde and Yalcin, 2016). These factors often cause postpartum depression (Figueiredo et al., 2013) and insufficient breastfeeding (Stuebe et al., 2014), which are associated with high incidence of premenopausal breast cancer, diabetes, and obesity in the mothers and autism, sudden death, and deficiency in maternal behaviors in their offspring (Ip et al., 2007).

Oxytocin (OT), a neuropeptide produced in hypothalamic supraoptic nucleus (SON), paraventricular nucleus (PVN) and several accessory neuroendocrine nuclei, not only plays a major role in baby delivery and milk ejection, but is also pivotal in maintaining maternal mental health (Hou et al., 2016). It has been reported that OT knockout mice show a failure of the milk-ejection reflex (MER) and thus cannot rear their offsprings (Lee et al., 2008). In virgin ovariectomized female rats, intracerebroventricular administration of OT can induce a rapid onset of full maternal behavior (Pedersen et al., 1982); the expression of OT receptor (OTR) is associated with variations in maternal behaviors (Francis et al., 2000). Thus, OTR signaling is essential for normal maternal behavior and lactation. By contrast, pup deprivation (PD) can disrupt the activity of OT neurons and the MER (Wang and Hatton, 2009b) while OT applied through nasal approach (IAO) can activate OT neurons in the hypothalamus (Liu et al., 2017), thereby having the potential to promote maternal health. In parallel with OTR signaling, as a chronic stress, PD can activate the hypothalamic-pituitary-adrenocortical (HPA) axis (Russell et al., 2018), which in turn inhibits OT neuronal activity (Di et al., 2005) and maternal behaviors (Pereira et al., 2015) and IAO likely reverses this process. However, effects of PD and IAO on interactions of the two systems remain unclear, particularly at the early stage of lactation.

To clarify the effect of IAO on maternal behavior and lactation and identify its underlying mechanisms, a rat model of intermittent PD (Wang and Hatton, 2009b; Liu et al., 2016) was used. We observed effects of IAO on PD-evoked depression-like behaviors and hypogalactia, and then analyzed the neuroendocrine mechanisms underlying IAO effect on PD-evoked postpartum depression and hypogalactia. The results allow us to better understand the underlying mechanisms and to propose novel approaches to improve maternal health.

## Materials and Methods

### Preparation of PD Model and IAO

Sprague-Dawley rats were used in this experiment. All the rats were housed and maintained on a 12–12 h light–dark cycle with free access to water and food. Two virgin females (180–250 g) and one proven breeder male (350–400 g) were housed in one cage for 1 week for making pregnant rats. The pregnant rats were randomly divided into control group, PD group and PD+IAO group. The PD model was made from lactating rats deprived 10 pups in the litter for 20 h/day from postpartum day 1 (PD1), continuously for 4 days (PD1 to PD4). All rat dams received 0.45% NaCl or 0.1 nmole OT in 0.45% NaCl nasally (Sigma-Aldrich, 10 μl for each naris), 3 times/day. During the separation from their biological mothers, the 10 pups in a litter were nursed by a foster mother. Control dams and pups received the same handling procedure as the PD dams did without the separation during observation. All the procedures were conducted in accordance with NIH Guidelines for the Care and Use of Animals and approved by the Animal Care and Use Committees of the Harbin Medical University.

### Observation of Maternal Behaviors

Maternal behaviors and lactation performance were evaluated on PD1 and PD4 before the brain, blood and mammary glands were sampled for further examinations next day. Items of observations included the latency of pup retrieval and latency of suckling for judging maternal behaviors; litter’s body weight gain (LBWG) was used for judging lactation efficiency. The whole procedure was video-taped for later analyses.

### Western Blots

Methods for processing proteins were modified from previous reports (Wang et al., 2013b; Liu et al., 2017). On PD5, protein expression and immunohistochemistry of the hypothalamus were analyzed without suckling. Dams were decapitated and the hypothalamus were removed and cooled down in ice-cold artificial CSF for 1–2 min. Then, the SON and PVN were isolated and homogenized in a RIPA lysis buffer (Yeasen, Shanghai, CAS#20115ES60). In brief, 30 μg protein per lane was separated on a 10% SDS-PAGE gel and then transferred onto polyvinylidenefluoride membrane. The protein membranes were incubated with TBS containing 5% dry milk (w/v) for 2 h at room temperature (21–23°C) and then incubated with primary antibodies (Santa Cruz Biotechnology, Shanghai) against glial fibrillary acidic protein (GFAP, SC6171, a marker of astrocytes), c-Fos (SC-7202), extracellular signal-regulated protein kinases (ERK) 1/2 (SC-514302), phosphorylated ERK (pERK) 1/2 (SC-136521, and Omnimabs, Shanghai, OM25780), corticotrophin-releasing hormone (CRH, SC-10718) and β-tubulin (Wanlei, Shanghai, WL-02296) in a dilution of 1:200 to 1:400 at 4°C for 12 h. ERK 1/2 and β-tubulin were used as loading controls. The protein membranes were further processed with horseradish peroxidase-conjugated secondary antibodies which matched with the species of corresponding primary antibodies and with an enhanced chemiluminescence detection kit (Tanon, Shanghai). Protein bands were visualized with an automated chemiluminescence imaging analysis system (Tanon 5200, Shanghai).

### Immunohistochemistry of the Hypothalamus

Methods of immunostaining were the same as previously described (Wang et al., 2013b; Liu et al., 2017). In brief, the hypothalamus was fixed in 4% paraformaldehyde for 24 h, and then cut using a vibratome into 60 μm thick sections that contained the SON. The sections were treated with 0.3% Triton X-100 for 60 min to permeabilize cell membranes and then with 5% bovine serum albumin for 60 min to block non-specific binding sites. After incubation with primary antibodies against GFAP, pERK 1/2, c-Fos (see above) and OT-NP (SC-393907) in a dilution of 1:200 at 4°C overnight, species-matched secondary antibodies (1:1000) were applied for 1.5 h at room temperature to label the corresponding primary antibodies. Lastly, Hoechst staining (0.5 μg/ml, 15 min) was used to label nuclei. Sections were first examined with a fluorescence microscope (Eclipse FN1, Nikon) through a CCD camera (DS Ri2, Nikon), results of which were further compared with the images taken with a confocal microscope (Thorlabs). To avoid false positive or negative results of immunostaining, serial dilutions of the primary antibody, staining with pre-absorbed primary antibody, no-primary and no secondary antibody controls were applied.

### Histochemistry of the Mammary Glands

In identification of the morphological features of mammary glands, conventional Hematoxylin and Eosin (H-E) staining was carried out after paraffin section (5 μm-thick) of the tissue. Images of the sections were taken using a Nikon microscopy and stored in computer for further analysis. To reduce the variability resulting from different loci, coronal sections of the middle part of the mammary glands from different groups were used for comparisons.

### Assay of Plasma Levels of Adrenocorticotropic Hormone (ACTH), Corticosterone, and OT

Assaying plasma levels of different hormones was performed using enzyme linked immunosorbent assay (ELISA, for ACTH, HY-10057; corticosterone, HY-10063; OT, HY-10017) or radioimmunoassay (for OT only) by Beijing Sino-UK Institute of Biological technology (Beijing). Briefly, dams were decapitated after observations and trunk blood (0.5 ml) was collected in heparinized tubes; the plasma was separated by centrifugation (3,000 rpm, 4°C, 15 min), and aliquots were stored at -20°C. ELISA experiment was performed using the following method. In brief, the standard was prepared in a series (4, 8, 16, 32, 64, and 128 pg/ml for ACTH; 10, 20, 40, 80, 160, 320, 640 ng/ml for corticosterone and 60, 30, 15, 7.5, 3.75, and 1.875 pg/ml for OT). Samples were thawed and warmed to room temperature for assays in duplicates. The wells of a 96-well plate were coated with 100 μl antibody solution in each well for 2 h; after rinsing with washing buffer and drying, 200 μl blocking buffer was added and kept for 30 min; after rinsing with washing buffer, shaking and drying, 50 μl standard solutions or sample solution (25 μl coating solution plus 25 μl plasma sample) were added to designed wells, respectively; then, 100 μl enzyme-labeled agent HRP solution was added to each well and kept for 60 min; after rinsing and drying, 100 μl color substrate solution was added and kept for 15 min for coloration before the reaction was stopped by adding 100 μl stopping solution. Optical density for each well was measured at 450 nm within 15 min of reaction stopping. To confirm the result of ELISA assay of OT levels, radioimmunoassay was performed with conventional method with the kit containing OT antibody (ab212193, Abcam, Shanghai) by the same company. The average recovery rates were >96% in all assays. The sensitivities for OT, ACTH and corticosterone were <0.9 ng/ml, <0.4 pg/ml, and <0.5 ng/ml, respectively. Intra-group and inter-group variations were less than 5 and 9%, respectively. Negative controls were set with diluted plasma and final results were based on the average results of duplicated samples.

### Data Collection and Analysis

Lactation failure was defined as a reduction in milk availability of more than 80% of the control. Signs of depression included those that showed loss of interests in the pups, such as delayed retrieval and suckling of pups (Wang et al., 2007). Data analyses of Western blots, and immunohistochemistry have been described in previous reports (Wang et al., 2013a). In brief, ImageJ or Photoshop was used for quantitation of protein bands (average luminosity multiply the total number of Pixels), which was further divided by the amount of their corresponding loading proteins; the protein amount of each bands in the control group was set as 1 or 100, and proteins in other groups were expressed as the fold or % of the control. In evaluation of confocal images, the fluorescence intensity in each channel was normalized to a standard curve (1–256) to allow for comparison between different experiments. The background level was set as 1 through minimum baseline correction. GFAP filaments were identified by fibrous GFAP positive staining that is an extension of astrocytic nucleus and longer than 20 μm (Wang and Hatton, 2009a). ANOVA, Kruskal–Wallis text or Wilcoxon rank test and χ^2^ test were used for statistical analyses where appropriate as instructed by SigmaStat program (SPSS, Chicago, IL, United States), and *P* < 0.05 was considered significant. All graphs were represented with box-whiskers with scattered plots indicating the median and the quartiles Q1 and Q3. All measures were also expressed as mean ± SEM in raw values or the percentage of controls.

## Results

In this study, we first tested the effect of PD and IAO on maternal behaviors and lactation efficiency in 63 dams and assayed plasma hormone levels. Then using Western blotting and immunohistochemistry, we identified activities of the OT-secreting system in 30 rats and assayed CRH contents in the PVN of 21 dams.

### Effect of IAO on PD-Evoked Maternal Behaviors and Hypogalactia

**Figure 1:**
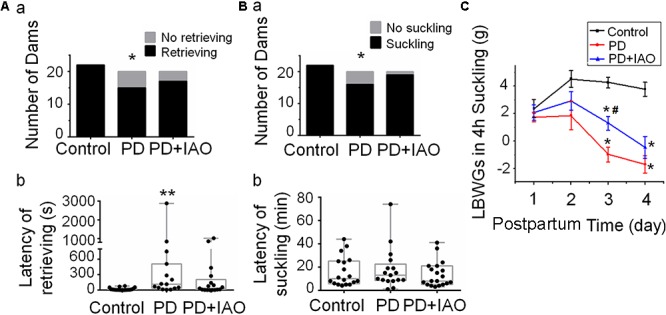
Effects of PD and IAO on maternal behaviors and lactation. **(A,B)** The number of dams’ retrieving **(Aa)** and suckling **(Ba)** their pups (black bars) or not (gray bars) during 1 h observation, and the average latency of dams’ retrieving **(Ab)** and suckling **(Bb)** their pups among dams retrieving and suckling pups. Graphs of **(Ab,Bb)** were represented with box-whiskers with scattered plots indicating the median and the quartiles. **(C)** The litter’s body weight gain (LBWG) in 4 h suckling throughout postpartum days 1–4 (PD1-4). PD, pup deprivation; IAO, oxytocin (OT) applied through nasal approach; ^∗^*P* < 0.05, ^∗∗^*P* < 0.01 compared with the control group; ^#^*P* < 0.05 compared with the PD group.

PD reduces suckling stimulation, causes maternal stress, and likely evokes aberrant maternal behaviors and hypogalactia. To test this hypothesis, we observed effects of PD on maternal behaviors and lactation performance following parturition. Before observation, rat dams were put into the observation cages and their pups were put back to the cage 30 min later. In general, PD dams showed reduced interest toward the litters with certain time-dependence and individual variation (Figure 1). That is, on PD1, all dams retrieved and suckled their pups; on PD4, the number of dams retrieving pups (15/20) was significantly smaller than that in the control group (22/22, *P* < 0.05 by Fisher exact test), which became insignificant after IAO (17/20, Figure 1Aa). Similar trend was present in the number of dams suckling the pups (22/22 in control group, 16/20 in PD group, and 19/20 in PD+IAO group) (Figure 1Ba). The same change was also present in the dams that retrieved and suckled their pups. The average latency of retrieving the pups on PD4 was significantly longer in the PD dams than the control dams [PD: 115 (34, 510), *n* = 15; Control: 6 (3,21), *n* = 22; Kruskal–Wallis test, *P* < 0.01]. However, IAO in PD dams shortened the latency significantly, which made the difference in the latency become insignificant between the control group and PD+IAO group [PD+IAO: 34 (3.5, 207.5), *n* = 17; Kruskal–Wallis test, *P* > 0.05] (Figure 1Ab). Correspondingly, IAO also removed the trend of elongated latency of suckling in the PD group, but there was no statistical significance among the three groups [control: 11.5 (5.8, 24.3), *n* = 22; PD: 11.5 (8.3, 19.0), *n* = 16; PD+IAO: 8 (5, 21), *n* = 19, Kruskal–Wallis test, *P* > 0.05] (Figure 1Bb).

In evaluation of the lactation efficiency, we found that changes in the intramammary pressure of the dams were not as sharp as what we have observed in the dams at the middle stage (days 8–12) of lactation under urethane anesthesia although a slow rising and falling in the pressure could be evoked by intravenous injection of 1 mU OT in control dams but rarely in the PD dams at day 4 (data not shown). Instead, we evaluated lactation efficiency by daily measuring LBWGs in 4 h suckling during dam-pup reunion throughout PD1∼4. The result showed that on PD3, LBWGs in the PD dams were significantly lower than that in the control group (4.25 ± 0.0.4 *g*, *n* = 6, in the control; -1 ± 0.5 g, *n* = 13, in the PD, *P* < 0.01), which was partially weakened in PD+IAO group (1.28 ± 0.5 *g*, *n* = 10, *P* < 0.01 compared with the PD group). On PD4, both the PD and PD+IAO remained significantly lower than the control in the LBWGs; however, the difference between PD and PD+IAO group disappeared (Figure 1C). By contrast, in control dams, IAO significantly shortened the latency of lactation and increased the duration of suckling the pups (Supplementary Figure S1).

### Effects of IAO on PD-Evoked Involution of Mammary Glands

**Figure 2:**
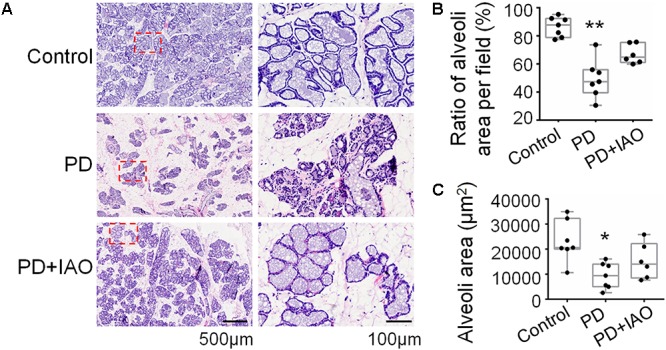
Effects of PD and IAO on the development of mammary glands. **(A)** HE staining of the mammary glands, the right panels are the enlargements of red squares in left panels. Box-whiskers with scattered plots summarizing statistical analysis of the ratio of alveolar area per field **(B)** and the average area in five biggest alveoli in one filed **(C)**. Others refer to Figure 1.

Upon pregnancy, the epithelium and lobule extensively proliferate and differentiate to meet the demand of milk production while weaning causes involution of them. The hypogalactia could be due to a failure of the milk production, secretion or ejection at the mammary glands. To test this hypothesis, we analyzed the histological feature of the mammary glands. Following 4 days of PD, relative to the enlarged and well-differentiated alveoli in control dams (Figure 2A), PD caused involution-like changes in the histology of the mammary glands. That is, the ratio between alveolar area and the total area of the mammary glands decreased [47.4% (39.6%, 56.0%) in PD vs. 87.8% (78.9%, 92.4%) in Control group, *n* = 7; Kruskal–Wallis test, *P* < 0.01]. The ratio remained lower in the PD+IAO group [64.9% (61.1%, 75.4%), *n* = 6, *P* > 0.05 compared with control group], but there was no statistically difference compared with PD groups, suggesting partial recovery.

Similarly, measuring the area of individual alveolar cavity revealed that PD dams [9413.1 (4985.1, 14029.5) μm^2^, *n* = 7] had significantly smaller alveolar cavity than that in the control dams [20582.1 (20006.2, 32243.2) μm^2^, *n* = 7, Kruskal–Wallis test, *P* < 0.05], and this difference became insignificant after IAO was applied to the PD dams [13993.9 (8445.1, 22183.0) μm^2^, *n* = 6, Kruskal–Wallis test, *P* > 0.05 to the control]. This result is in agreement with the incomplete recovery of the depression-like behaviors and the LBWGs.

### Effects of PD and IAO on Cellular Activities in the SON

**Figure 3:**
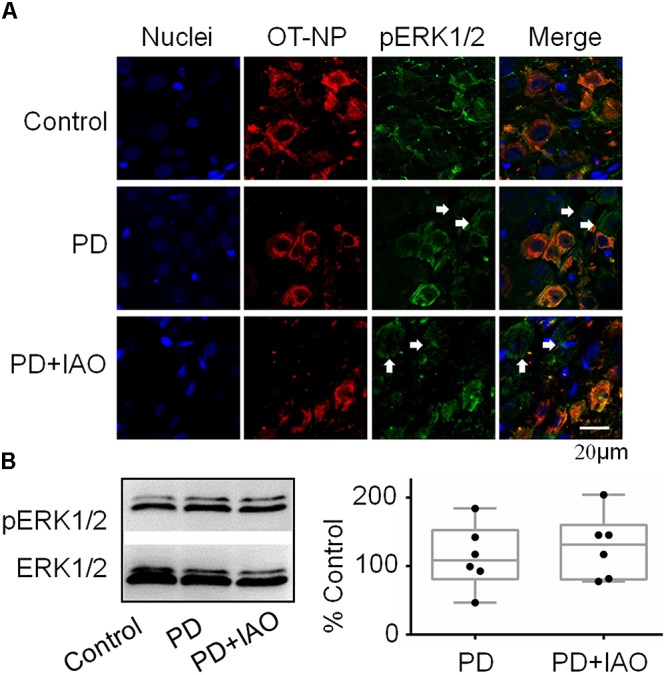
Effects of PD and IAO on the expressions of pERK1/2 in the SON. **(A)** Fluorescent microscopy of immunostaining images of nuclei (in sky blue), OT-neurophysin (OT-NP, in red), pERK1/2 (in green), and their merges. The arrows point to non-OT neurons. **(B)** Western blot bands (left panel) and the summary graphs (right panel) showing the expression of pERK1/2 under different experimental conditions. Annotations are the same as Figure 1.

The improving effect of IAO on maternal behaviors and lactation performance suggests involvement of the CNS, particularly the OT-secreting system, as suggested by the study on PD effects on maternal behaviors and lactation in the middle stage of suckling (Liu et al., 2016). To test this hypothesis, we first observed the expression of pERK 1/2, a marker of cellular activation in the SON (Wang and Hatton, 2007b), in OT neurons by immunohistochemistry (Figure 3A). The result showed that pERK 1/2-positive SON neurons were 29.3 ± 6.8 in control (*n* = 6), 35.3 ± 11.3 in PD (*n* = 4) and 32.5 ± 8.3 in PD+IAO (*n* = 5), respectively. By contrast, the ratio of neurons/pERK 1/2-positive neurons was 0.91 ± 0.10 in control (*n* = 6), 0.64 ± 0.10 in PD (*n* = 4) and 0.70 ± 0.07 in PD+IAO (*n* = 5), respectively. This result highlights a trend of reduction in pERK 1/2-positive OT neurons in PD and PD+IAO groups, which accompanied with an increase in pERK 1/2-positive vasopressin (VP) neurons, the only neuron that co-exists with OT neurons in the SON.

Moreover, we assayed protein levels of pERK1/2 in the SON following the 4 day PD. In Western blotting, there was no significant difference in the level of pERK 1/2 between the control and PD groups [108.3 (81.0, 152.6) % of the control, *n* = 6, Kruskal–Wallis test, *P* > 0.05]. Similar to PD group, the level of pERK 1/2 in the PD+IAO group showed an insignificant increase [131.2 (80.5, 160.1) % of the control, *n* = 6, Kruskal–Wallis test, *P* > 0.05] (Figure 3B).

**Figure 4:**
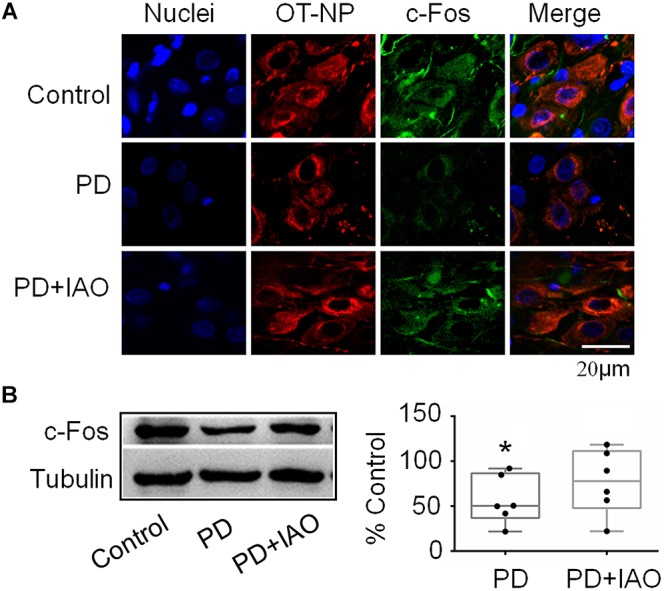
Effects of PD and IAO on the expressions of c-Fos in the SON. **(A)** Fluorescent microscopy of immunostaining images of nuclei (in sky blue), OT-neurophysin (OT-NP, in red), c-Fos (in green), and their merges. **(B)** Western blotbands (left panel) and the summary graphs (right panel) showing the expression of c-Fos under different experimental conditions. Annotations are the same as Figure 1.

By contrast, c-Fos expression in the SON decreased significantly in the PD group, which was partially blocked in the PD+IAO group in the immunohistochemical observation (Figure 4A). This finding was confirmed in Western blot analysis. As shown in Figure 4B, PD significantly decreased the protein levels of c-Fos [50.3 (36.8, 86.6) % of the control, *n* = 6, Kruskal–Wallis test, *P* < 0.05], which recovered partially in the PD+IAO group [77.8 (48.0, 111.3) % of control, *n* = 6, Kruskal–Wallis test, *P* > 0.05 compared to the control group]. As a strong marker of OT neuronal activation (Fenelon et al., 1993), the reduction in PD and the recovery in PD+IAO in c-Fos expression are consistent with the changes in maternal behaviors and lactation performance.

### Effects of PD and IAO on Astrocytic Plasticity in the SON

**Figure 5:**
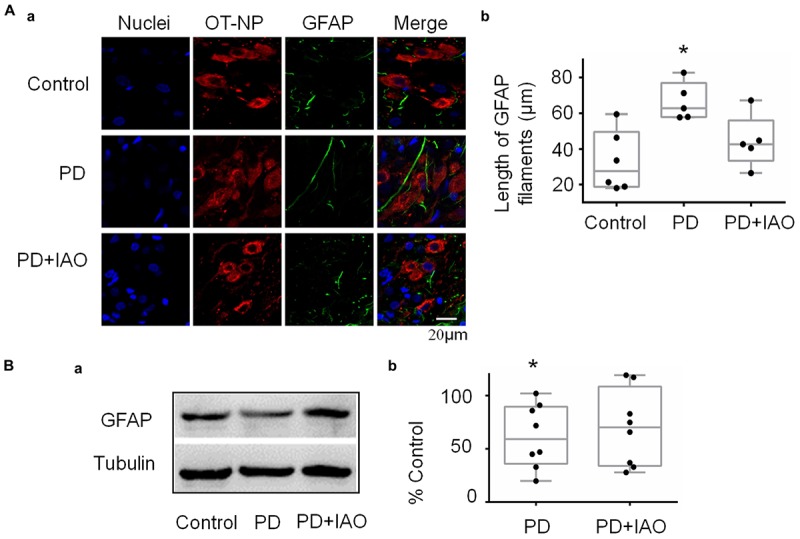
Effects of PD and IAO on GFAP plasticity in the SON. **(A)** Fluorescent microscopy of immunostaining of GFAP filaments. From left to the right, the images show nuclei, OT-NP, GFAP, and their merges **(Aa)**; the right panel exhibiting the statistical result of average length of GFAP filaments **(Ab)**. **(B)** Western blot bands **(Ba)** and the summary graphs **(Bb)** showing the expression of GFAP under different experimental conditions. Annotations are the same as Figure 1.

There is a close interaction between neurons and astrocytes in activities of the OT-secreting system (Hou et al., 2016). PD-evoked alteration in OT neuronal activity could also result from aberrant astrocytic plasticity that is usually represented by GFAP, a cytoskeletal and scaffolding protein of astrocytes (Wang and Parpura, 2016). Thus, we observed GFAP plastic changes under PD without and with IAO (Figure 5). The result in immunohistochemistry showed that the length of GFAP filament increased significantly [62.8 (57.9, 77.0) μm, *n* = 5, Kruskal–Wallis test, *P* < 0.05] in PD compared with the control group [27.5 (18.7, 49.7) μm, *n* = 6]. With IAO, the elongation of GFAP filament attenuated [42.8 (33.5, 56.0) μm, *n* = 5, Kruskal–Wallis test, *P* > 0.05]. The representative images of fluorescent microscopy are shown in Figure 5A.

Corresponding to the morphological observation, we also analyzed levels of GFAP protein expression in the SON. As shown in Figure 5B, PD significantly reduced the level of GFAP monomers [59.7 (36.3, 89.5) % of control, *n* = 8, Kruskal–Wallis test, *P* < 0.05 compared to the control] and PD+IAO insignificantly reversed this trend [70.5 (34.0, 108.7) % of control, *n* = 8, Kruskal–Wallis test, *P* > 0.05]. Supplementary Figure S2 shows a full panel of the blots in Figure 3B, 4B and 5B.

### Effects of PD and IAO on Hormone Levels in the Plasma

**Figure 6:**
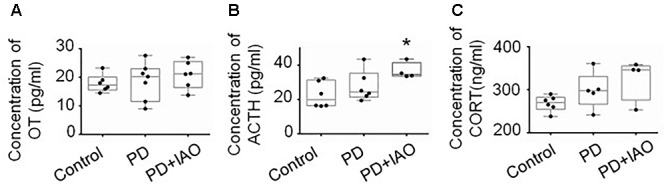
Effects of PD and IAO on plasma levels of OT, ACTH, and corticosterone. **(A)** OT concentration. **(B)** Levels of ACTH. **(C)** Levels of corticosterone (CORT). Annotations are the same as Figure 1.

Maternal behaviors and lactation are all associated with the production of OT; the depression-like behavior and hypogalactia possibly result from reduced OT secretion. To test this hypothesis, we assayed OT concentration in plasma with ELISA and then again using radioimmunoassay (Figure 6A). Unexpectedly, no obvious difference in the three groups was identified in the plasma although there was a trend of increased OT levels following PD and PD+IAO [Control: 17.3 (15.5, 20.1) pg/ml, *n* = 6; PD: 20.2 (11.5, 23.0) pg/ml, *n* = 7; PD+IAO: 21.2 (16.4, 25.5) pg/ml, *n* = 6, Kruskal–Wallis test, *P* > 0.05]. This is in agreement with the knowledge that OT functions during lactation are mainly at its pulsatile but not tonic pattern of secretion/action.

On the other hand, PD is a kind of chronic stress and unavoidably influences the activity of HPA axis. Thus, we assayed plasma concentration of stress hormones, ACTH, and corticosterone levels by ELISA. The results showed that PD+IAO evoked significant increase in ACTH [34.6 (33.6, 41.4) pg/ml, *n* = 4 in PD+IAO vs. 20.0 (16.5, 31.3) pg/ml, *n* = 6 in control, Kruskal–Wallis test, *P* < 0.05]. But in corticosterone, there was no difference [270.7 (254.7, 282.7) ng/ml, *n* = 6 in control group; 297.7 (266.9, 330.8) ng/ml, *n* = 5 in PD group; 346.1 (276.6, 355.6) ng/ml, *n* = 4 in PD+IAO group, Kruskal–Wallis test, *P* > 0.05] (Figure 6B,C). However, in an average level, PD+IAO did significantly increase corticosterone levels compared to the control (326.1 ± 24.3 ng/ml in BD+IAO vs. 268.4 ± 7.4 ng/ml in control, *P* < 0.05 by ANOVA).

### Effects of PD and IAO on CRH Levels in the PVN

**Figure 7:**
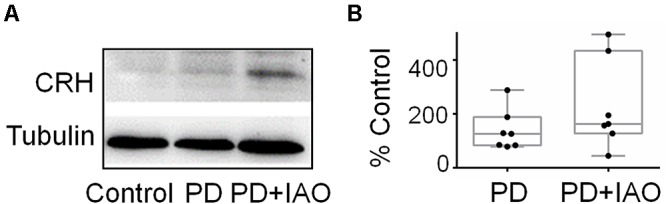
Effects of PD and IAO on CRH expression in the PVN. **(A)** Western blot bands. **(B)** The summary graphs. Annotations are the same as Figure 1.

Increases in plasma ACTH and corticosterone levels suggest activation of the HPA axis, which is mainly assumed due to activation of CRH neurons in the PVN. To examine if IAO effect is also associated with CRH expression, we assayed the expression of CRH in Western blot (Figure 7). The result showed that PD and PD+IAO tended to increase CRH levels, particularly in PD+IAO dams (230.6 ± 63.2% of the control, *n* = 7, *P* < 0.05 by ANOVA); however, these did not reach statistically significant levels with non-parametric statistical analysis [126.0 (83.6, 187.9) % of the control, *n* = 7, *P* > 0.05 in PD; 162.6 (127.1, 433.7) % of the control, *n* = 7, in the PD+IAO group].

## Discussion

The present studies revealed that PD can cause postpartum depression-like maternal behaviors and hypogalactia with dramatic time dependence and certain individual variation. These effects are associated with reduced activity of the OT-secreting system, particularly astrocytes-associated inhibition of pulsatile OT secretion and early involution of the mammary glands for the MER. IAO can partially reverse these PD-evoked psycho-physical changes. However, activation of the HPA axis likely compromises the improving effects of IAO on PD-evoked changes. These findings highlight both the therapeutic potential of IAO in postpartum depression and hypogalactia and the challenges of integrative strategy of improving maternal health.

### Effects of PD and PD Plus IAO on Maternal Behaviors and Lactation

Pup deprivation dams experience physical and psychological stress, which can be mimicked by the rat model of intermittent PD (Liu et al., 2016). It has been reported that maternal attachment behaviors and representations declined linearly with the duration of mother–infant separation (Feldman et al., 1999); early life stress due to maternal separation can induce anxiety- and aggressive-like behavior during adolescence (Shin et al., 2016). However, effects of PD on maternal behaviors have not been studied extensively. The present study revealed direct effects of PD on maternal behaviors of the dams. As presented in Figure 1, PD significantly reduced the ratio of retrieving and suckling pups while elongating the latency of pup retrieval by the dams that exhibited retrieval behaviors during observation, indicating reduced interest in pups, a critical sign of maternal depression (Wang et al., 2007). This finding is in agreement with a previous report that PD disrupted lactation while causing maternal depression in the rat dams at the middle stage of lactation (Liu et al., 2016) as well as clinical observations that OT can improve the mother’s mood and the relationship between mother and baby (Mah et al., 2013; Mah, 2016) and that OT can mitigate the depressive-like behaviors of maternal separation stress through modulating mitochondrial function and neuroinflammation (Amini-Khoei et al., 2017).

Accompanying with the maternal depression, hypogalactia occurred in PD dams. As shown in Figure 1, PD reduced the LBWGs gradually during the 4 h dam-pup reunion, which became statistically significant on PD3 and PD4. Obviously, PD influence on lactation performance is associated with the length of separation, which not only causes the recession of the hypothalamic machinery for the MER, but also results in early involution-like changes in the histology of the mammary glands. As shown in Figure 2, PD dams had significantly smaller area of alveoli and increased fatty interstitial tissues compared to the control dams. Since we could not count all the alveoli, the increased fatty tissues did not exclude the possibility of reduction in total number of alveoli and thus, fully evaluating the features of PD-evoked changes in the mammary glands are needed. However, current finding of the histological alteration in the mammary glands could account for the hypogalactia, at least partially.

Different from the effect on maternal behaviors, IAO enhanced the LBWGs in PD dams, particularly on PD3; however, this effect is short-lasting since there was no obvious effect of IAO on the PD4. Noteworthy is that plasma OT levels in the PD dams did not decrease compared to the control levels with or without IAO. Thus, the hypogalactia is likely a result of failure of the burst firing pattern of OT neurons and losses of pulsatile OT secretion that directly determine the milk ejection as previously identified in the middle stage of lactation (Wang and Hatton, 2009b; Liu et al., 2016). This finding is in agreement with the report of Fewtrell and colleagues that OT nasal spray in mothers with preterm babies could induce initial faster milk production but then convergence between groups (Fewtrell et al., 2006).

### Effects of PD and IAO on Cellular Activities in the OT-Secreting System

OT can influence mental activity in the brain and milk letdown at the mammary glands by OT release from the hypothalamo-neurohypophysial system. The SON is a main source of OT in the brain and the blood (Hou et al., 2016). Therefore, we examined the cellular activity of the SON in PD dams. We found that PD significantly decreased the expression of c-Fos in the SON (Figure 4) although there was no significant change in pERK1/2 expression (Figure 3), an indicator of instant firing activity of OT neurons (Wang and Hatton, 2007b). An important contributor for the inhibition of OT neuronal activity is maladapted astrocytic plasticity. As shown in Figure 5, there was an expansion of GFAP filament in PD dams, suggesting expansion of astrocyte processes that is known as a key factor inhibiting OT neuronal activity (Wang and Hamilton, 2009; Wang and Zhu, 2014). Together with the previous finding (Liu et al., 2016), we can conclude that PD suppresses the activity of OT neurons and in turn reduces OT release into the brain and blood, leading to postpartum depression and hypogalactia.

In general, the effect of IAO on PD dams was similar to that on control dams (our unpublished data), i.e., increased the expression of both pERK 1/2 and c-Fos. In PD dams, IAO also tended to increase the expression of pERK 1/2 while made the reduction in c-Fos levels insignificantly different from the control. As a general inhibitory factor, the retraction of GFAP and its associated astrocyte processes can account for the weakened reduction of c-Fos, allowing OT neurons to regain its activity in response to suckling. As for the mechanisms underlying reduction in the expression of GFAP monomers, we currently do not have evidence to explain; however, different spatiotemporal distribution of pERK 1/2 and protein kinase A (Wang et al., 2017) could be a reason, which remains to be identified under PD condition.

### Effects of PD and IAO on the Activity of HPA Axis

The possibility that HPA axis can be activated by chronic stress (Russell et al., 2018) is further tested in the present study. We found that PD tended to increase the expression of CRH in the PVN in Western blots (5/7 rats showed an increase in CRH levels) as well as plasma ACTH and corticosterone levels although that did not reach a statistical significant levels due to big variation of one or two samples. Thus, the maternal depression and hypogalactia were not likely a result of tonic release of OT into the blood as evidenced in assaying plasma OT levels but possibly due to interrupting the burst firing ability of OT neurons by potentially increased activity of the HPA axis (Pereira et al., 2015), which should be verified in future study.

What come out unexpectedly are the increased plasma levels of ACTH and corticosterone by IAO in PD dams as well as CRH expression in the PVN. Appropriate and stable plasma corticosterone level is one of the essential factors for dams to take care of the offspring (Hasiec and Misztal, 2018); however, chronically increased corticosterone level could inhibit excitatory input on OT neurons (Di et al., 2005) and decreased maternal behaviors (Pereira et al., 2015), particularly a pulsatile pattern (Wang and Hatton, 2004, 2007a), thereby inhibited burst generation in OT neurons and the MER.

The increased activity of the HPA axis likely resulted from a general activation of both the SON and PVN including CRH neurons. As exhibited in the result, the expression of c-Fos became significantly high following IAO in PD dams involving OT neurons, VP neurons and likely astrocytes in the SON. The SON and PVN share many common structural and functional features and connect closely in the MER and thus, it is reasonable to believe that the two nuclei under went the same activation process during PD and following the IAO. Thus, CRH neurons could be activated in this process although that did not reach a statistical significant level due to big variation of one or two samples following the non-parametric analysis. We have to say that using Western blot to study on CRH expression allows only hemi-quantification; RIA or ELISA seems a better approach. Hopefully, a more trustable supplier would make all the assays more accurate in future study.

Alternatively, the activation of HPA axis could result from increased VP neuronal activity. As shown in Figure 3, PD+IAO also increased the number of VP neurons with pERK 1/2 staining, which reflects increased VP neuronal activity despite that could be differentiated in Western blotting. VP is generally believed to be an important activator of the ACTH cells and the HPA axis (Yayou et al., 2007). Thus, its activation should partially account for the increased plasma ACTH and the subsequently increased plasma corticosterone levels following IAO. In addition, because lactating rats exhibited a high degree of CRH and VP colocalization in parvocellular PVN neurons, hypothalamic projections, and median eminence terminals compared to virgins, it is possible for a simultaneous increase in VP and CRH in the PVN (Milewski et al., 2016).

## Conclusion

The improving effect of IAO on the maternal health could be compromised by the simultaneous activation of HPA axis in association with potentially increased glutamatergic input from the olfactory bulbs (Hatton and Wang, 2008). Moreover, IAO can partially improve the activity of the OT-secreting system and prevents aberrant maternal behaviors, although it is not enough to reverse the reduced milk availability in PD dams due to the early involution-like alteration in the histology of the mammary glands. Thus, while using IAO, peripheral application of OT in a pulsatile manner (Liu et al., 2016) is also necessary to maintain the normal response of the MER and to reduce the risk of premenopausal breast cancer.

## Author Contributions

XL, DL, TL, and HL collected data. XL and DL analyzed data. XL wrote the first draft. Y-FW designed the study and made the final revision. All authors participated in discussion and revision.

## Conflict of Interest Statement

The authors declare that the research was conducted in the absence of any commercial or financial relationships that could be construed as a potential conflict of interest.

## Supplementary Material

Figure S1 Effects of IAO on the latency and duration of suckling in control dams. **(A)** Latency; **(B)** duration. Other annotations refer to Figure 1.

Figure S2 Full blots showing the effect of PD and PD+IAO on the expression of pERK1/2, c-Fos and GFAP proteins in Figure 3–5, respectively. Other annotations refer to Figure 1 and 3.
