# Supplementary figures and images for: Effects of Intranasal Oxytocin on Pup Deprivation-Evoked Aberrant Maternal Behavior and Hypogalactia in Rat Dams and the Underlying Mechanisms
Source: Front Neurosci. 2019 Feb 26;13:122. doi: 10.3389/fnins.2019.00122 (PMC6399306; doi:10.3389/fnins.2019.00122)

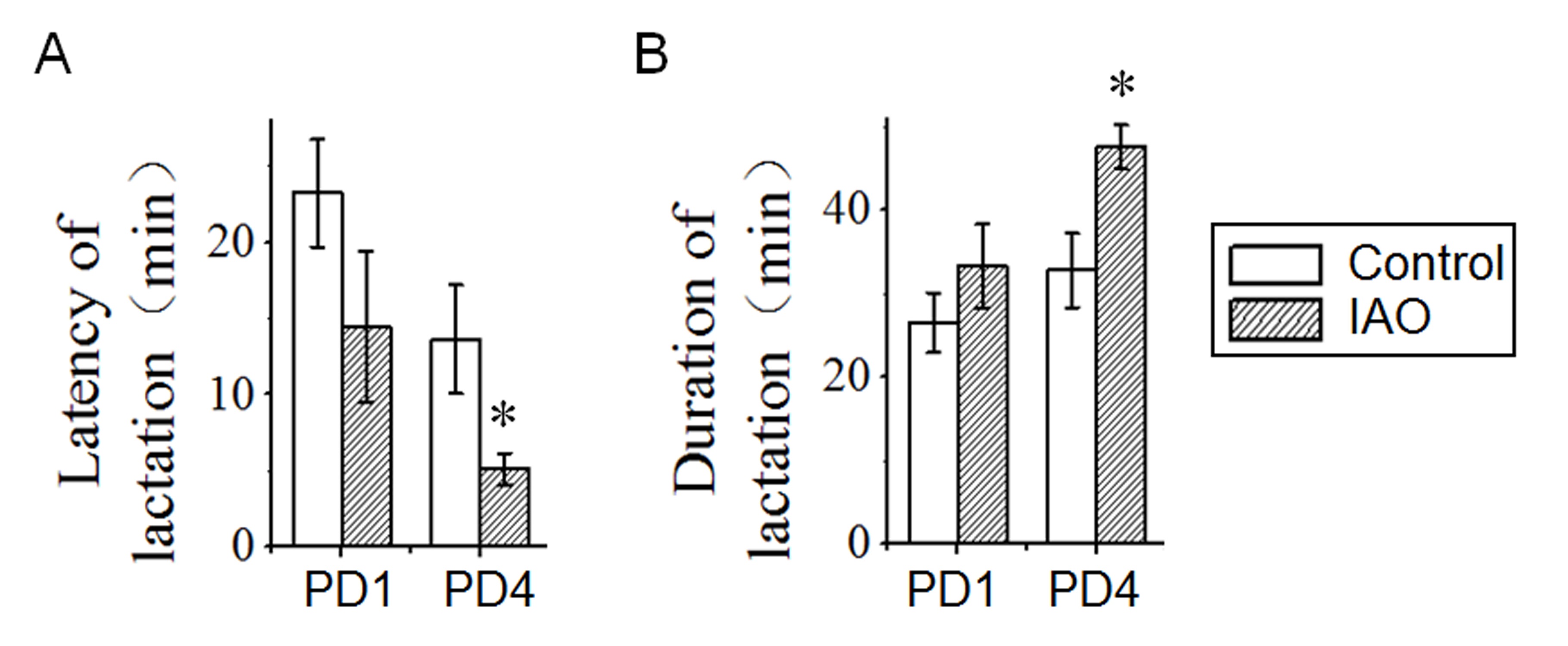

Supplement: Figure S1 — Effects of IAO on the latency and duration of suckling in control dams. (A) Latency; (B) duration. Other annotations refer to Figure 1. [file Image_1.jpg]

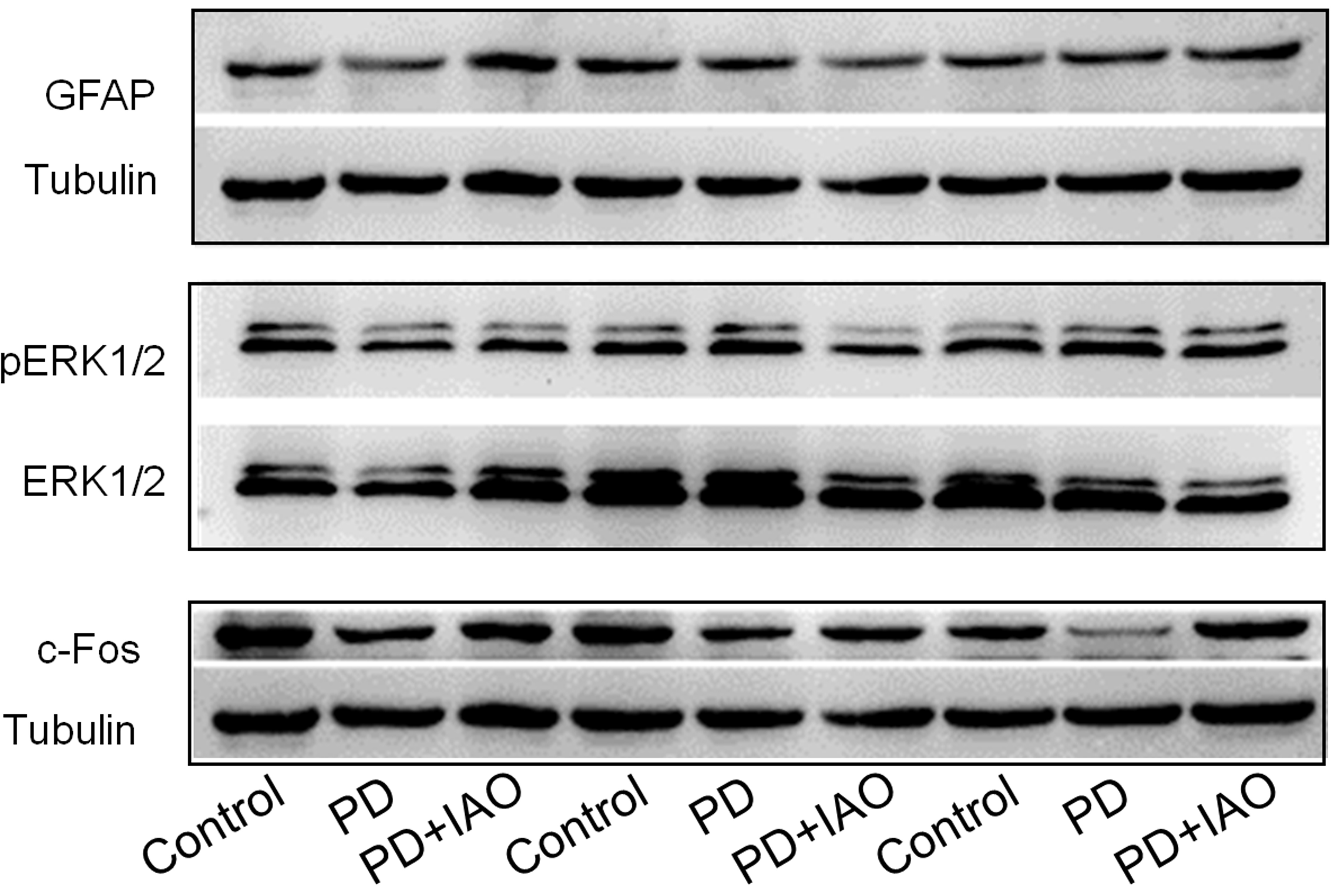

Supplement: Figure S2 — Full blots showing the effect of PD and PD+IAO on the expression of pERK1/2, c-Fos and GFAP proteins in Figure 3–5, respectively. Other annotations refer to Figure 1 and 3. [file Image_2.jpg]
